# Supplementary material for: Automated Nuclear Cartography Reveals Conserved Sperm Chromosome Territory Localization across 2 Million Years of Mouse Evolution
Source: Genes (Basel). 2019 Feb 1;10(2):109. doi: 10.3390/genes10020109 (PMC6409866; doi:10.3390/genes10020109)
Supplement: Supplementary file 1 [file genes-10-00109-s001.zip › genes-427841 supp material ready to publish.pdf]

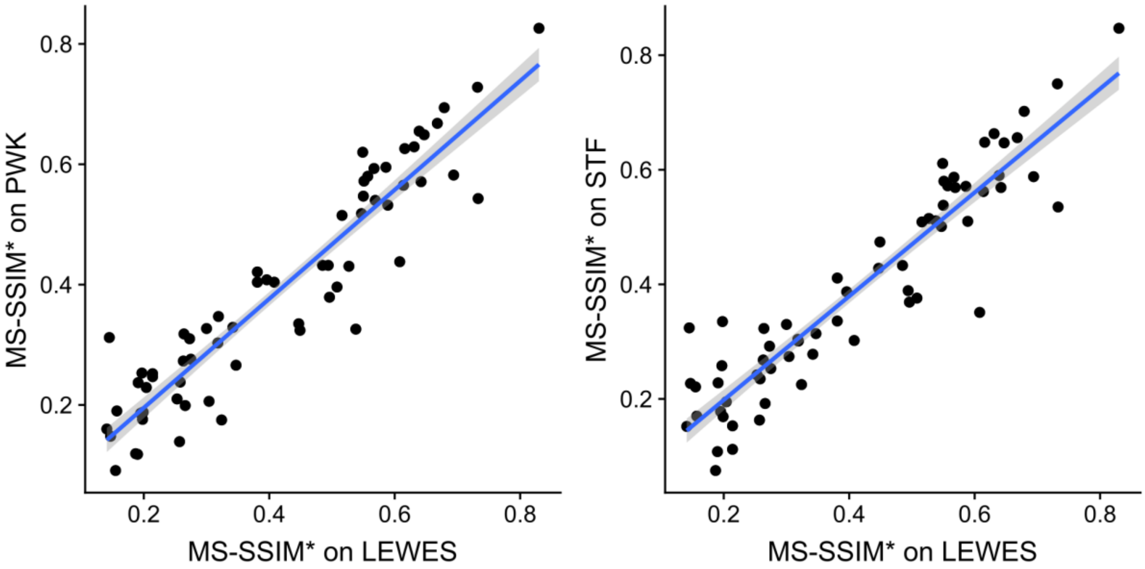

Figure 1. Chromosomes X and 19 co-hybridization.

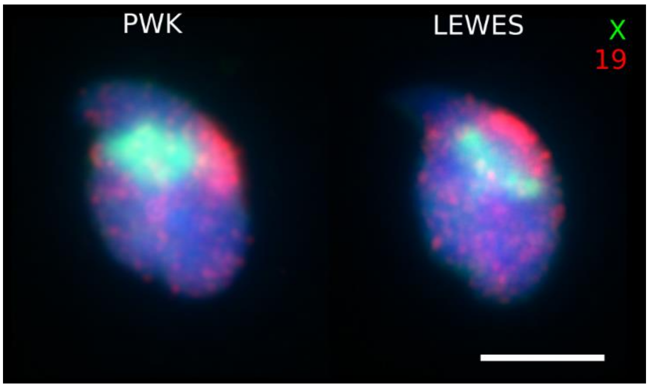

Figure 2. Comparison of MS-SSIM * scores using different warping templates.

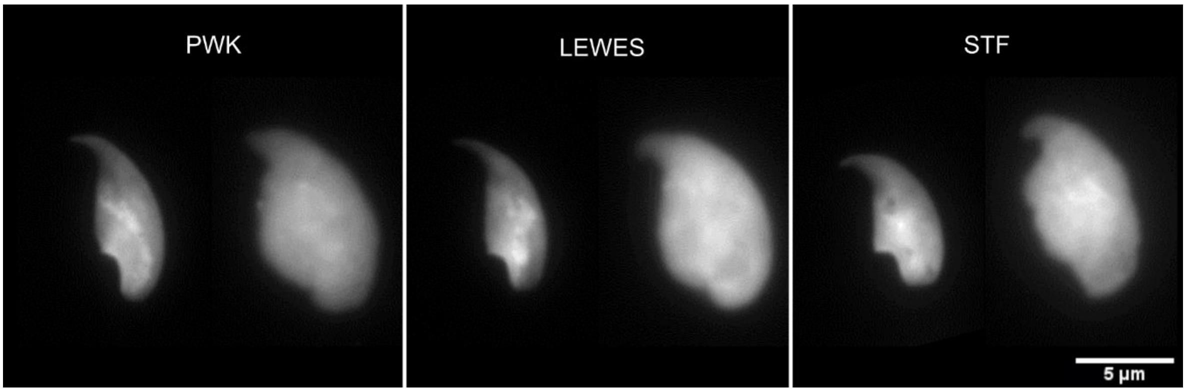

Figure 3. Examples of swelled and unswelled nuclei.
